# Anti-MOG IgG in EAE models clinical aspects of pediatric MOGAD

**DOI:** 10.3389/fimmu.2026.1860892

**Published:** 2026-06-15

**Authors:** Yike Jiang, Estefany Y. Reyes, Elliot YH. Lin, Emily C. Troutman, Miranda Lumbreras, Devon T. DiPalma, Heather Van Mater, Mari L. Shinohara

**Affiliations:** 1 Division of Pediatric Rheumatology, Department of Pediatrics, Duke University School of Medicine, Durham, NC, United States; 2 Department of Integrative Immunobiology, Duke University School of Medicine, Durham, NC, United States; 3 Department of Molecular Genetics & Microbiology, Duke University School of Medicine, Durham, NC, United States; 4 Department of Cell Biology, Duke University School of Medicine, Durham, NC, United States; 5 Department of Neurobiology, Duke University School of Medicine, Durham, NC, United States

**Keywords:** autoantibody, autoimmune, demyelination, EAE, MOGAD, pediatric

## Abstract

**Introduction:**

Myelin oligodendrocyte glycoprotein (MOG) antibody-associated disease (MOGAD) is a severe, autoantibody-mediated neuroinflammatory syndrome that disproportionately impacts children. Detection of conformation-specific anti-MOG IgG1 in the serum is central to MOGAD diagnosis, yet the pathogenic role of these antibodies remains unclear. We sought to develop a clinically informed model of pediatric MOGAD to study the immunopathogenesis that recapitulates 1) early age of disease onset, 2) anti-MOG IgG1 in serum, and 3) widespread inflammation in the CNS.

**Methods:**

We employed two approaches to model MOGAD using anti-MOG IgG in mice: 1) treating young C57BL/6 mice with the murine-derived monoclonal MOG antibody 8-18C5, and 2) using young IgH^MOG^ transgenic (Tg) mice, engineered to express the immunoglobulin heavy chain of 8-18C5. These mice were induced with experimental autoimmune encephalomyelitis (EAE) by immunizing with MOG_35–55_ to elicit inflammatory demyelination.

**Results:**

Both exogenous and endogenous anti-MOG IgG exacerbated EAE disease in young mice. Compared to wild-type littermates (+/+), young IgH^MOG^ Tg mice (Tg/+) exhibited reduced peripheral immune cells and greater neutrophil-to-lymphocyte ratios. Tg/+ mice also had increased CNS leukocyte infiltration compared to wild-type littermates. Tg/+ mice developed monophasic circumferential longitudinal myelitis, bilateral optic neuritis, and multifocal brain inflammation. Innate immune cells in the CNS, including microglia, showed significant downregulation of surface FcγRII/III. This downregulation on microglia was accompanied by enhanced uptake of cells expressing MOG, suggesting Fc receptor-mediated internalization of MOG immune complexes.

**Discussion:**

Introduction of anti-MOG IgG in EAE recapitulates key aspects of pediatric MOGAD and enables dissection of the mechanisms underlying anti-MOG antibody-mediated immunopathogenesis.

## 1 Introduction

Myelin oligodendrocyte glycoprotein antibody-associated disease (MOGAD) is an acquired demyelinating syndrome that disproportionately affects pediatric patients (1–3). The clinical presentation of MOGAD is highly heterogeneous, including optic neuritis, myelitis, acute disseminated encephalomyelitis (ADEM), and cerebral cortical encephalitis, rendering all parts of the CNS vulnerable to inflammation. The first proposed criterion was published in 2018 (4), around the same time that conformation−specific assays detecting anti−MOG IgG1 in serum became clinically available (5). Since then, MOGAD has been recognized as a distinct clinical entity from other autoimmune demyelinating conditions like multiple sclerosis (MS) and neuromyelitis optica spectrum disorder (NMOSD) (4). Unlike these other autoimmune demyelinating conditions, MOGAD is more likely to impact children. Approximately 30% of MOGAD cases occur in the pediatric age group (6) and among children with acquired CNS demyelinating syndromes, MOGAD comprises 40% of cases (7, 8). Moreover, MOGAD had a threefold higher incidence in children than in adults in a Dutch study (9), although epidemiology data are still forthcoming. Furthermore, children less than 10 years old have higher titers of anti-MOG antibodies compared to older age groups and have more severe disease with brain involvement compared to adults (10–12). Diagnosis of MOGAD relies on the serological detection of anti−MOG IgG1, which binds conformational epitopes of MOG and can only be detected using conformationally sensitive cell−based assays (4). Human anti−MOG antibodies have been shown *in vitro* to mediate demyelination through several mechanisms, including complement activation and Fc receptor-dependent cytotoxicity (13, 14). However, how anti-MOG antibodies operate *in vivo* within the full neuroimmune environment remains incompletely understood. Therefore, there is a critical need to develop clinically informed models that capture both the early age of onset and the presence of anti-MOG antibodies.

We sought to develop mouse models of pediatric MOGAD to study immunopathogenesis that recapitulated important clinical features of the disease: early age of disease onset, anti-MOG IgG1 in the serum, and susceptibility of all CNS regions to inflammation. To do so, we leveraged existing tools: a rodent anti-MOG antibody, clone 8-18C5 (15), and the transgenic (Tg) IgH^MOG^ mouse strain, in which the endogenous Ig heavy chain gene locus is replaced with the heavy chain sequence of 8-18C5 (16). Prior literature on 8−18C5 and IgH^MOG^ mice anticipated the pathogenic potential of anti−MOG antibodies, emerging well before the clinical recognition of MOGAD in humans. For instance, the injection of 8-18C5 during EAE exacerbates the disease (17), and crossbreeding IgH^MOG^ mice with 2D2 mice (transgenic mice with MOG-specific T cell receptors) results in spontaneous EAE (18, 19). Although the latter was initially believed to model Devic’s disease, a term now retired and understood to fall within the NMOSD, it more closely resembles MOGAD in retrospect. Importantly, neither 8-18C5 injection alone nor IgH^MOG^ mice develop clinical disease without MOG immunization, suggesting the anti-MOG IgG alone is insufficient to cause MOGAD (3, 16, 20, 21). Previous studies have used these tools to model MOGAD in specific contexts, such as evaluating effects on the visual system (21) and testing the therapeutic potential of inhibiting antibody recycling (22). Our study applied broader evaluations to characterize the CNS pathology of anti-MOG IgG during EAE using immunophenotyping and histological approaches. In contrast to most prior EAE studies that rely on adult mice to model adult−onset MS, we deliberately used young mice to reflect pediatric−onset MOGAD. Young mice are generally more resistant to EAE induction than adult or aged mice (23, 24). We therefore selected post-weaning 4-5-week-old mice to avoid the confounding effects of breastfeeding and to better approximate the neurodevelopmental stage of pediatric patients. Mice at this age exhibit incomplete myelination, active synaptic pruning, and ongoing cerebellar maturation, features that roughly correspond to human childhood and adolescence (25, 26).

Our models revealed notable similarities with human MOGAD and new insights into the pathogenesis of anti-MOG IgG. In particular, we identified antibody-mediated microglial phagocytosis of MOG through Fc receptors as a novel activity of anti-MOG IgG.

## 2 Materials and methods

### 2.1 Mice

C57BL/6J mice were purchased from The Jackson Laboratory (Strain # 000664) and bred in-house. IgH^MOG^ was generously provided by Dr. Gregory Wu (Washington University, USA) with the permission of Dr. Hartmut Wekerle (Max-Planck-Institut, Germany). IgH^MOG^Tg/+ corresponds to heterozygous animals with the knock-in transgene, while +/+ denotes wild-type littermates without the transgene. Both male and female mice were used in our experiments; no differences in results were found between the sexes. Young mice were defined as 4–5 weeks old at the time of MOG_35–55_ immunization. Mice were euthanized in carbon dioxide inhalation chambers, followed by decapitation or cardiac perfusion and organ removal as secondary methods. Mice were maintained and bred in the pathogen-free animal facility at Duke University. All animal experiments were performed as approved by the Institutional Animal Care and Use Committee (IACUC) at Duke University.

### 2.2 Cell lines

HEK293T cells were kindly provided by the laboratory of Dr. Ashley Moseman (Duke University). HEK293T cells were grown in DMEM supplemented with 10% FBS (Atlas Biologicals) and 1% penicillin-streptomycin (Thermo Fisher Scientific). BV2 murine microglia cells were kindly provided by the laboratory of Dr. Cagla Eroglu (Duke University). BV2 cells were grown in DMEM supplemented with 10% FBS, 1% penicillin-streptomycin, and 2mM L-glutamine (Thermo Fisher Scientific).

### 2.3 Antibodies:

#### 2.3.1 Murine anti-MOG IgG1 (8-18C5) and isotype control

8-18C5 was purchased from GenoVac with the assistance of Dr. Anke Salmen (Ruhr-University, Germany). InVivoMAb mouse IgG1 isotype control, clone MOPC-21, was purchased from BioXcell.

#### 2.3.2 Flow cytometry:

**Table:** 

Antigen	Fluorophore	Concentration	Company	Clone	Cat
CD11b	BV605	1:400	BioLegend	M1/70	101257
CD16/32	BV421	1:200	BioLegend	93	101331
CD16/32	APC-Cy7	1:400	BioLegend	93	101327
CD19	PE	1:500	BioLegend	6D5	115508
CD4	APC-Cy7	1:500	BioLegend	GK1.5	100414
CD4	PE-Cy7	1:200	BioLegend	GK1.5	100422
CD45	BUV395	1:200	BD Biosciences	30-F11	564279
CD8α	PE-Cy5	1:500	BD Biosciences	53-6.7	553034
CD8α	APC-Cy7	1:200	BioLegend	53-6.7	100714
IFNγ	AF488	1:200	BioLegend	XMG1.2	505813
IgG1	APC	1:200	BioLegend	RMG1-1	406609
IL-17A	PE	1:200	BioLegend	TC11-18H10.1	506904
Ly6C	BV711	1:500	BioLegend	HK1.4	128037
Ly6G	AF700	1:500	BioLegend	1A8	127622
NK1.1	PE-Cy7	1:500	BioLegend	PK136	108714
TCRβ	FITC	1:500	BioLegend	H57-597	109205
TCRβ	PE-Cy5	1:200	BioLegend	H57-597	109210

### 2.4 EAE induction and scoring

Mice were anesthetized with 4% isoflurane in oxygen delivered via vaporizer for EAE induction. On day 0, 100 μg of mouse MOG35–55 peptide (United Biosystems) was emulsified in Incomplete Freund’s Adjuvant (Sigma) containing 50 μg per mouse of heat-killed *Mycobacterium tuberculosis* (BD Sciences) and subcutaneously injected as previously described (27, 28). Experiments did not use Pertussis toxin (PTx, List Biological Laboratories), unless noted otherwise. When PTx was used, we intraperitoneally injected 200 ng per mouse on days 0 and 2. PTx was omitted from the experiments presented in the main figures because we found that it was not required to elicit a robust disease phenotype in our models. In addition, prior studies have shown that PTx can inhibit B cell function *in vitro* (29) and *in vivo* (30, 31). In the indicated experiments, 200 μg of 8-18C5 or IgG1 isotype control was injected retro-orbitally, similar to a previous article (22), on day 5. EAE is scored as follows: 0 – no disease, 0.5 – partial tail limpness, 1 – tail limpness, 1.5 – impaired righting reflex, 2 – partial hindlimb paralysis, 2.5 – partial hindlimb paralysis with dragging of at least one hind paw, 3 – bilateral hindlimb paralysis, 3.5 – severe bilateral hindlimb paralysis partial forelimb paralysis, 4 – quadriplegia, 5 – moribund/death.

### 2.5 Confocal microscopy on CNS tissue sections

Following euthanasia, transcardiac perfusion was performed on mice with a minimum of 10 mL of PBS containing 0.1% heparin (Sagent Pharmaceuticals), followed by 10 mL of 2% paraformaldehyde. Brains, spinal cords, and optic nerves were extracted and fixed in 4% PFA overnight at room temperature, followed by dehydration in 30% sucrose for 1–3 days at 4 °C. Afterward, the CNS tissues were embedded in Tissue-Tek OCT compound (Sakura) for cryostat sectioning to obtain 20-micron sections. Spinal cord and brain sections were stained with FluoromyelinTM Red (Thermo Fisher Scientific), DAPI, and goat or donkey anti-mouse IgG AlexaFluor488 (Thermo Fisher Scientific) to detect myelin, nuclei, and IgG, respectively. Optic nerves were stained whole after overnight permeabilization with PBS plus 0.1% Triton X-100 (Sigma-Aldrich). Images were taken with an Andor Dragonfly Spinning Disk Confocal PLUS system (Andor Technology). Stitched images were captured using a Nikon CFI Plan Apochromat 10X/0.45 NA air objective, and high-magnification images were obtained with a Plan-Apochromat 63X/1.47 NA oil objective. Fluorescence was excited with solid-state lasers at 405, 488, 561, and 637 nm, and detected with an Andor Zyla 4.2 PLUS sCMOS camera. Acquisition was managed using Andor Fusion software (v2.4). Images were acquired at 2048 × 2043 pixels with a pixel size of 0.65 μm. Bandpass emission filters were used as follows: DAPI (447/60 nm), Alexa 488 (525/50 nm), FluoromyelinTM Red (600/50 nm). Data were analyzed on Imaris and FIJI ImageJ.

### 2.6 Flow cytometry

Brains, spinal cords, and optic nerves were harvested from euthanized mice and minced for 1 minute using small scissors, digested in 1X PBS containing 5% heat-inactivated fetal bovine serum (Atlanta Biologicals), 1mM HEPES (Thermo Fisher Scientific), Collagenase Type IV (Roche), and DNase I (Millipore) for 30 minutes at 37 °C with constant shaking at 225 rpm. Following the enzymatic digestion, single-cell suspensions were prepared by passing the cells through a 23-gauge needle and then filtering through a 70-μm cell strainer. Cells were then resuspended in 38% Percoll (Cytiva) and centrifuged at 532g for 30 minutes without braking. Following centrifugation, the lipid and debris layers were aspirated from the top of the tube, and the pelleted cells were hemolyzed and resuspended in staining buffer. Only freshly isolated cells were used for all flow cytometry analysis. All cell suspensions were thoroughly washed and immunolabeled for flow cytometry. Live/Dead Zombie UV^TM^ (BioLegend) was used to exclude dead cells for analyses. Spectral flow cytometry was performed using a Cytek Aurora. Blood was collected by facial vein bleed into heparinized tubes following 4% isoflurane in oxygen induction, and flow cytometry was performed immediately after.

### 2.6 Cytokine intracellular staining for flow cytometry

Single-cell suspensions at 2-3 × 10^6^ per well were stimulated in 96-well U-bottom plates with 100 ng/mL PMA and 375 ng/mL ionomycin for 4 h at 37 °C in the presence of 5.0 µg/ml Brefeldin A (BioLegend). Then, the cells were washed twice with PBS and stained with Live/Dead^TM^ Fixable Violet (BioLegend) and Fc block for 10 min at 4 °C. Surface marker staining was performed for 30 min at 4 °C without cell permeabilization. Subsequently, cells were fixed and permeabilized using Fixation Reagent and Perm/Wash Buffer (BioLegend) for 30 min at 4 °C, followed by intracellular cytokine staining with antibodies for 30 min at 4 °C. Flow cytometry was performed using a BD FACSCanto-II.

### 2.8 Quantification of conformation-specific anti-MOG IgG1

We employed a live cell-based assay to quantify conformation-specific anti-MOG IgG1, similar to clinically available tests (32, 33). Briefly, HEK293T cells were transfected with murine MOG-turboGFP (OriGene, MG203093) to express cell surface MOG. Transfected cells were then incubated with serum samples. Cell-bound IgG1 was detected using an APC-conjugated anti-IgG1 secondary antibody (**Supplementary Figure S1A**). Flow cytometry was performed using a BD FACSCanto-II. Correlation between anti-MOG IgG1 concentrations and APC signal intensity on turboGFP-positive cells (**Supplementary Figure S1B**). APC mean fluorescence intensity (MFI) of turboGFP-negative cells, representing non-specific binding, was subtracted from the MFI of the turboGFP-positive cells, as described previously (32). The relationship between subtracted APC MFI and anti-MOG IgG1 concentration is illustrated in the standard curve (**Supplementary Figure S1C**).

### 2.9 Live animal MRI

Mice were anesthetized with isoflurane at an initial concentration of 4%. After positioning on a custom mouse cradle, the concentration was reduced to 1.5% for maintenance. Body temperature was maintained at 37 °C using a circulating warm-water bath in the mouse cradle, and respiration was monitored throughout the scan. T2-weighted images were acquired with a Bruker BioSpec 70/20 MRI system equipped with an AVIII console and 90mm gradient coils (RRI, Billerica, MA). RF transmission was performed using a 72 mm quadrature volume coil (Rapid Biomedical, Rimpar, Germany), and a 4-channel mouse brain surface coil was used for signal reception (Bruker Biospin, Ettlingen, Germany). The multi-slice multi-echo sequence was chosen to reduce artifacts and sample multiple echo times. Imaging parameters were: field of view = 19.2 x 19.2 x 15mm, matrix = 128 x 128, 30 slices, echo spacing = 7 ms, and echo train length = 10. Individual echo images were averaged to improve signal-to-noise and T2 contrast.

### 2.10 Antibody-dependent phagocytosis assay

HEK293T cells were transfected with murine MOG-turboGFP (OriGene, MG203093) as phagocytosis targets. Then, transfected cells were pre-incubated with murine anti-MOG IgG1 (8-18C5) and co-cultured with BV2 murine microglia prelabeled with Tag it-Violet™ (Biolegend) as effector cells. Target and effector cells were co-cultured at a 1:1 ratio overnight. Cells were stained with CD16/32. Phagocytosis was measured as the percentage of turboGFP-positive BV2 cells. Dead cells (7-AAD positive) were excluded from analyses. Flow cytometry was performed using a BD FACSCanto-II.

### 2.11 Statistical analysis

Data analysis was performed in Spectroflow version 3.3.0, FlowJo version 10.1, and Prism GraphPad version 10.6.1. Unpaired t-tests were used, and *p* values ≤ 0.05 were considered significant.

## 3 Results

### 3.1 Exogenous anti-MOG IgG1 augments EAE in young mice.

**Figure 1:**
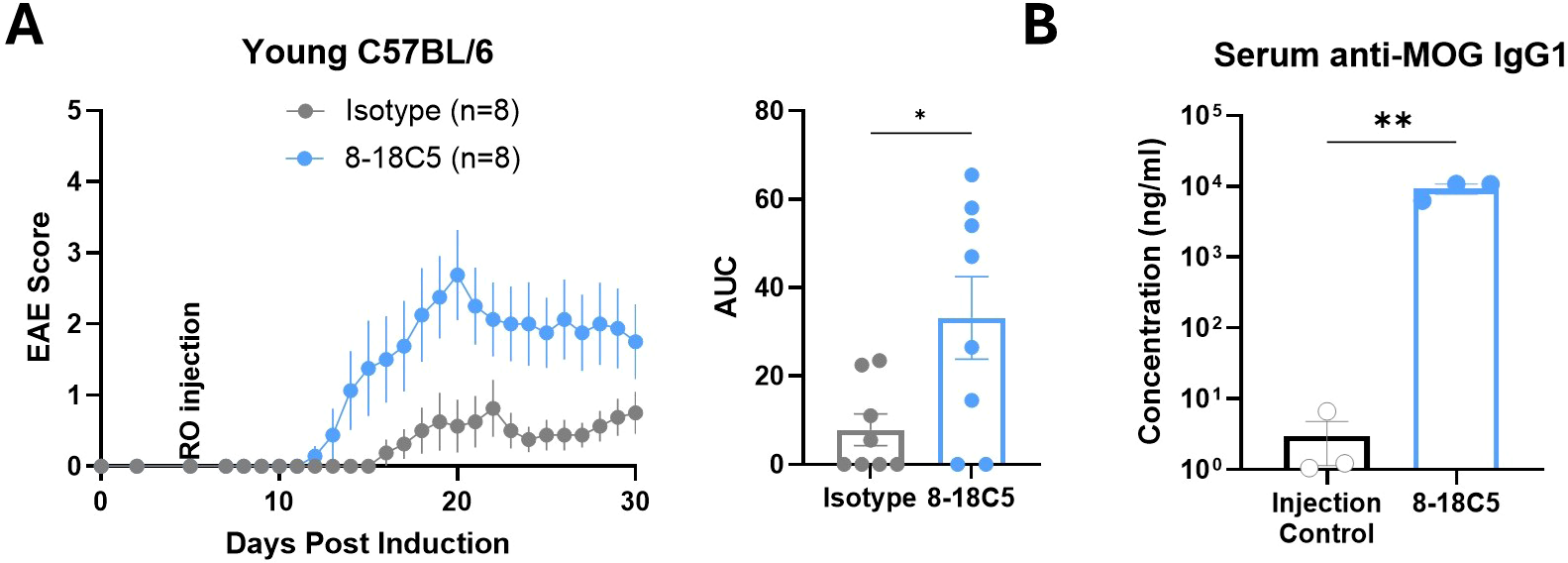
Anti-MOG IgG1 augments EAE disease in young mice. **(A)** 4-5-week-old C57BL/6 mice were immunized with MOG_35–55_ without PTX. At day 5, mice were injected retro-orbitally with 200 μg of 8-18C5 versus an isotype control. Lines represent the mean EAE disease score with SEM. Unpaired t-tests were used. **(B)** Serum anti-MOG IgG1 at endpoint was measured by flow cytometric cell-based assay. The data represent one of three similar experiments. Unpaired t-tests were used. *P ≤ 0.05, **P ≤ 0.01.

We first recapitulated the original study showing that anti-MOG IgG1 injections augment EAE disease (17), but with young C57BL/6 mice, intending to model pediatric-onset illness. We induced EAE without using pertussis toxin and administered anti-MOG IgG (clone 8-18C5) on day 5. In young mice, EAE disease was more severe with 8-18C5 (**Figure 1A**). To detect conformation-specific anti-MOG IgG1, we used a flow cytometric cell-based assay similar to the clinical test as described in the methods. This cell-based approach was necessary because linearized epitopes do not capture clinically significant human anti-MOG antibodies (34, 35). Using this method, 8-18C5 was still detectable in the serum even at day 34 post-induction (**Figure 1B**). We also observed that conformation-specific anti-MOG IgG1 is not produced by MOG_35–55_ immunization alone.

### 3.2 Endogenous anti-MOG IgG augments EAE in young IgH^MOG^ mice

**Figure 2:**
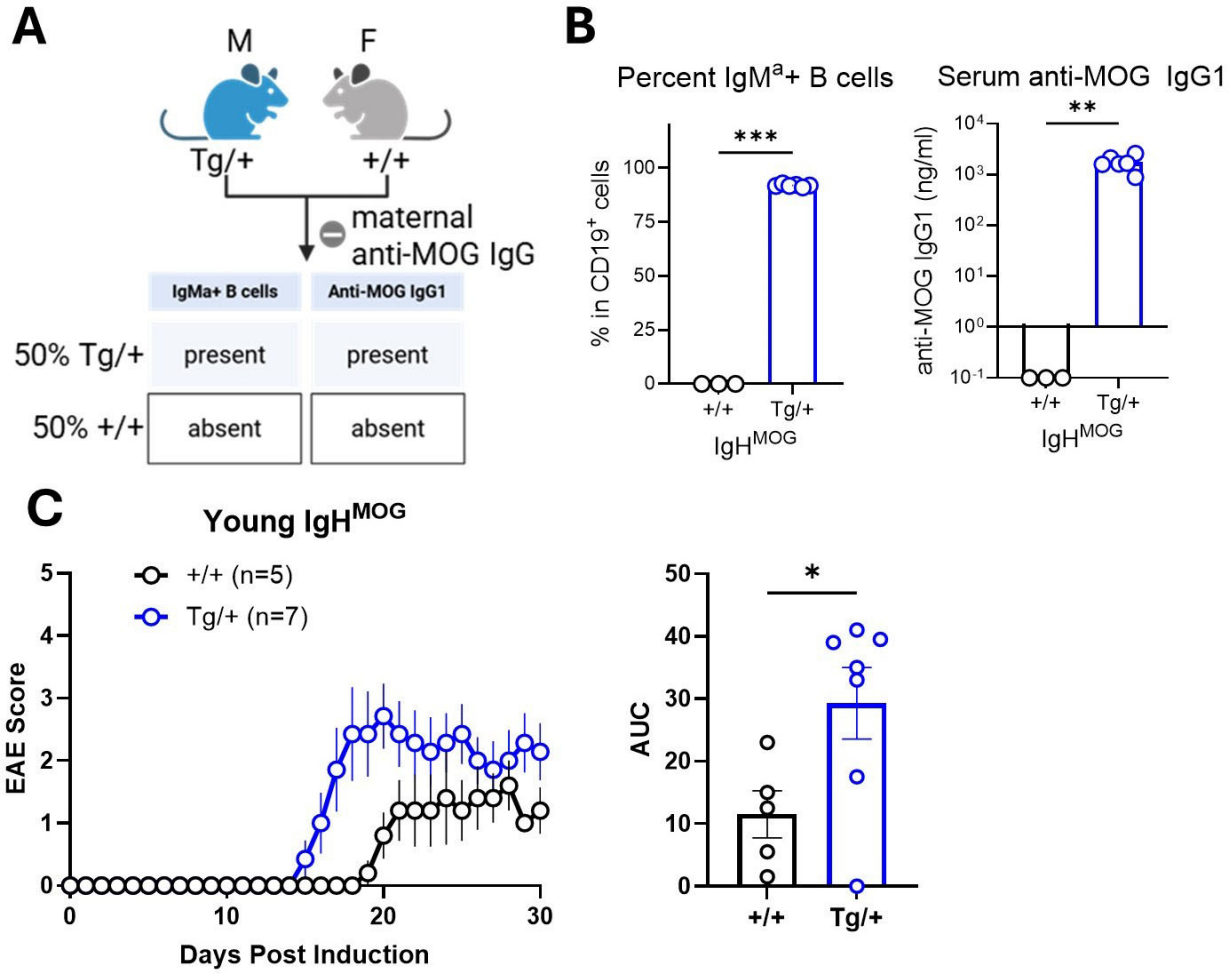
EAE disease is exacerbated in young IgH^MOG^ mice. **(A)** Breeding scheme with a heterozygous male IgH^MOG^ Tg/+ mouse crossed with a wild-type C57BL/6J female mouse. **(B)** Proportions of blood B cells with congenically marked Tg B cells (left), and serum anti-MOG IgG1 levels at weaning (right). **(C)** 4-5-week-old progeny mice were immunized with MOG_35–55_ without PTX. Lines represent mean EAE disease scores with SEM. The data represent one of three experiments. Unpaired t-tests were used. *P ≤ 0.05, **P ≤ 0.01, ***P ≤ 0.001.

We then sought to evaluate EAE disease with endogenously produced anti-MOG IgG. The knock-in transgenic (Tg) IgH^MOG^ mouse line expresses the rearranged heavy chain (IgH) sequence of the 8-18C5 clone in the germline J_H_ locus (16). As a result, the majority of B cells in IgH^MOG^ mice express a B cell receptor that recognizes MOG, and these Tg B cells are marked with IgM^a^ (16). In these experiments, using IgH^MOG^ sires for breeding was essential because we found that maternal antibodies contribute to EAE disease in the progeny. In the breeding scheme between Tg/+ (IgH^MOG^ heterozygous) breeders (**Supplementary Figure S2A**), 25% of the progeny are +/+ (homozygous of the wild-type genotype) and did not inherit the transgene (**Supplementary Figure S2B**). However, maternal anti-MOG antibodies were transferred to +/+ progeny (**Supplementary Figure S2C**), and +/+ mice showed EAE disease scores similar to those of Tg/Tg (transgene homozygous) and Tg/+ mice (**Supplementary Figure S2D**). Based on this finding, we relied on a breeding strategy of outbreeding IgH^MOG^ Tg/+ sires with wild-type C57BL/6 dams (**Figure 2A**) to avoid the interference from maternal antibodies (**Figure 2B**). Tg/+ progeny exhibited more severe EAE disease, peaking at days 14-16, compared to wild-type littermate controls (**Figure 2C**).

### 3.3 Reduced peripheral immune cells in IgH^MOG^ mice during EAE

**Figure 3:**
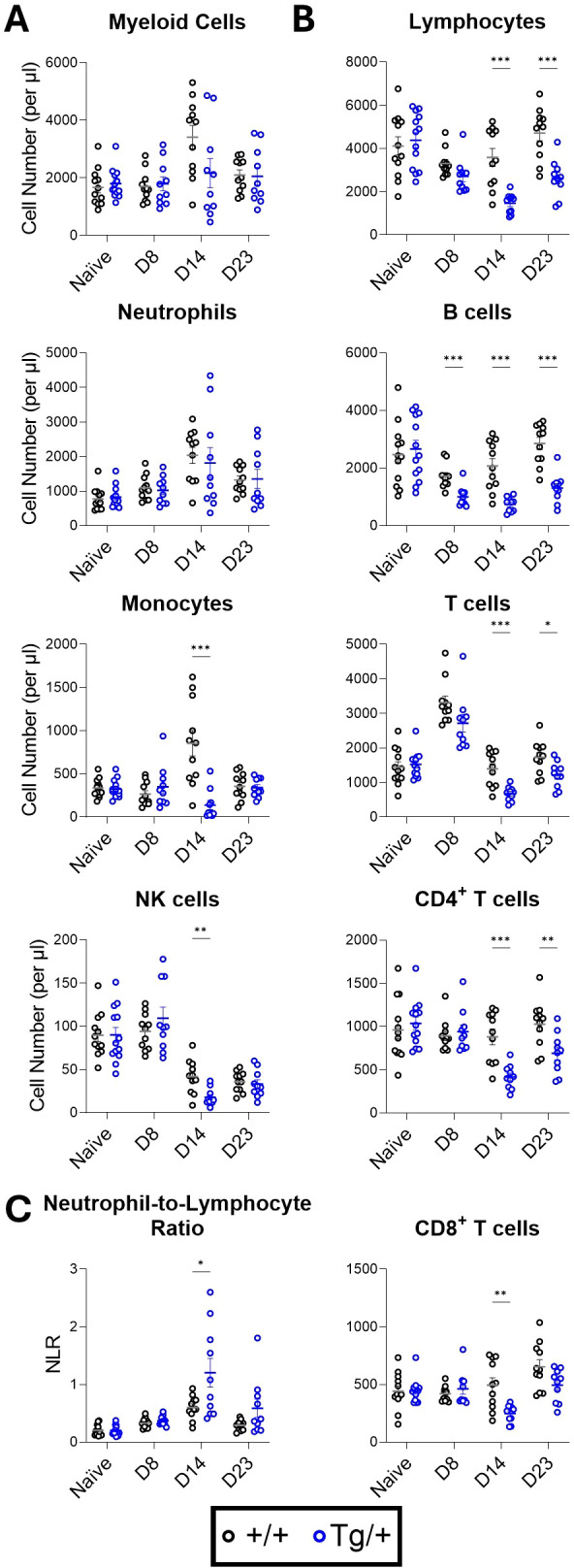
Reduced peripheral immune cells in IgH^MOG^ mice. Mice were immunized with MOG_35–55_ without PTX as described in the methods, using 4-5-week-old IgH^MOG^ mice. **(A, B)** Blood was collected on days 8, 14, and 23 post-induction, and compared to naïve mice, and flow cytometry subsetted innate **(A)** and adaptive **(B)** immune cells. Gating schemes are displayed in **Supplementary Figure S3A**. **(C)** Neutrophil-to-Lymphocyte ratio was derived from blood flow cytometry. Individual animals are represented as points with SEM. Unpaired t-tests were used for comparisons within each timepoint. *P ≤ 0.05, **P ≤ 0.01, ***P ≤ 0.001.

Next, we aimed to immunophenotype peripheral immune cells outside the CNS during EAE using the IgH^MOG^ Tg mice. We performed flow cytometry on serial blood draws and found significant changes in the circulating innate (**Figure 3A**) and adaptive immune subsets (**Figure 3B**). Among the myeloid population, we observed a decrease in monocytes at day 14 post-EAE induction in Tg/+. We also observed a reduction of lymphocytes, including NK cells, B cells, CD4^+^ and CD8^+^ T cells, at day 14 in Tg/+, compared to +/+ littermate controls. The Neutrophil-to-Lymphocyte Ratio (NLR) is known to be elevated during acute MOGAD attacks and correlates with Expanded Disability Status Scale (EDSS) scores clinically (36), so we investigated the NLR in MOG-immunized mice and found a significantly elevated NLR at day 14 (**Figure 3C**).

### 3.4 IgH^MOG^ mice develop circumferential longitudinal myelitis

**Figure 4:**
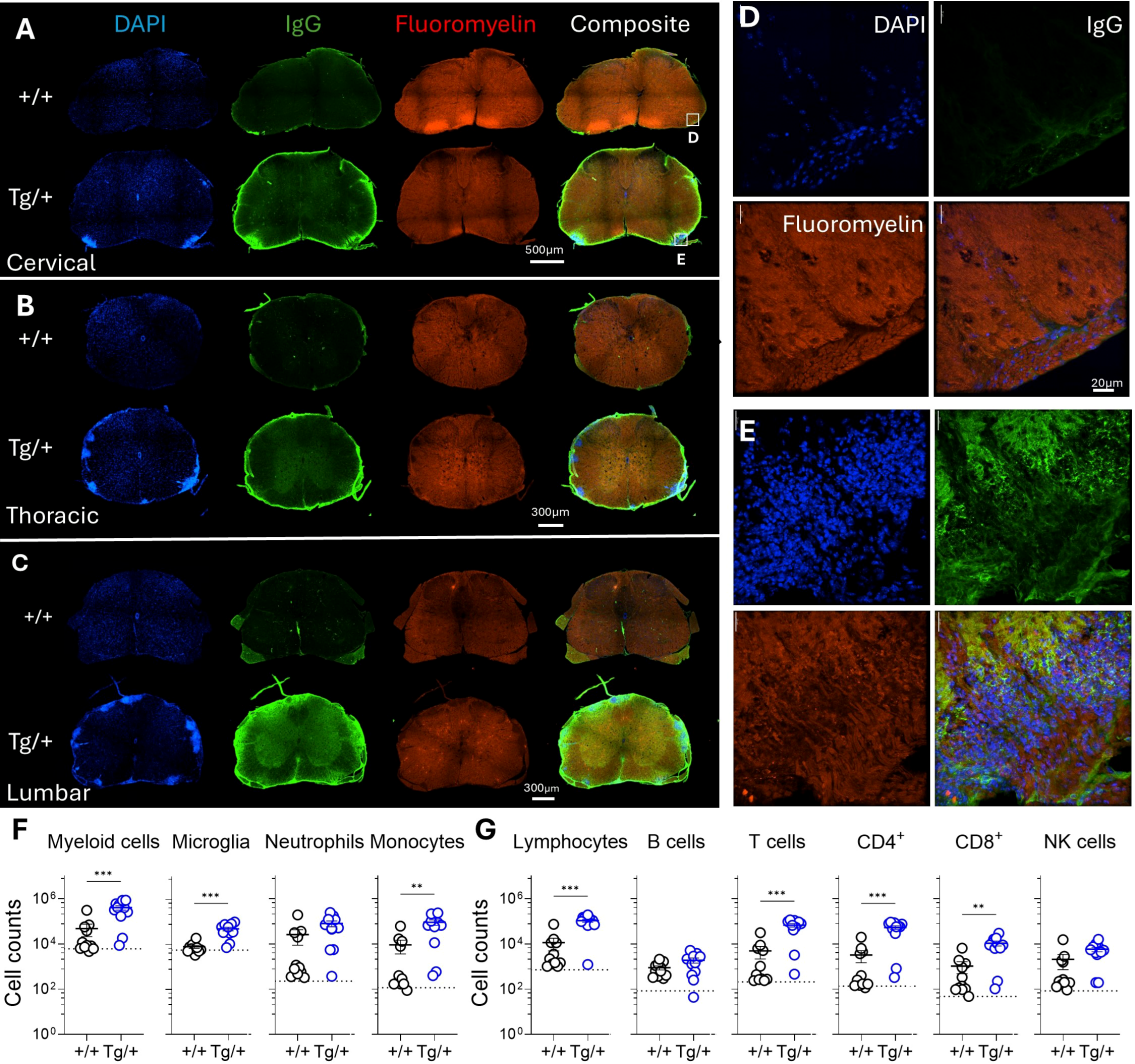
Longitudinal myelitis in IgH^MOG^ mice. Five-week-old IgH^MOG^ mice were immunized with MOG_35–55_ without PTX. **(A-E)** Confocal microscopy images showing transverse sections of the cervical **(A)**, thoracic **(B)**, and lumbar **(C)** spinal cord at day 14. DAPI is blue, IgG is green, and Fluoromyelin stain is red. The boxed areas were imaged at 63X and displayed to the right **(D, E)**. **(F, G)** Flow cytometry of whole spinal cord cells at day 15 after MOG_35–55_ immunization, showing total cell count per spinal cord. The gating scheme is displayed in **Supplementary Figure S3B**. Proportions of leukocyte subsets in CD45^+^ cells are displayed in **Supplementary Figure S4A**. The dotted line represents values from naïve IgH^MOG^ mice. The data represent one of three similar experiments. Individual animals are represented as points with SEM. Unpaired t-tests were used. **P ≤ 0.01, ***P ≤ 0.001.

We next qualitatively examined IgG distribution and demyelination in the CNS, starting with the spinal cord. On day 14, we found multiple foci of DAPI staining located circumferentially around the cervical (**Figure 4A**), thoracic (**Figure 4B**), and lumbar (**Figure 4C**) spinal cord sections in the Tg/+ animals compared to the +/+ wild-type littermates. We also found circumferential IgG staining in the Tg/+ mice that was absent from the +/+ controls. At higher magnification of images from Tg/+ mice, the peripheral foci of cell nuclei staining corresponded to areas of disrupted myelin (**Figures 4D, E**). We then used flow cytometry to quantify leukocyte infiltration in the spinal cords (**Figures 4F, G**). Compared to +/+ littermates, Tg/+ mice showed a significantly increased number of myeloid (**Figure 4F**) and lymphoid cells (**Figure 4G**), particularly in microglia, monocytes, CD4^+^ and CD8^+^ T cells at day 15. Interestingly, the total number of B cells was not different. Significant increase in CD4^+^ T cell frequency in Tg/+ mice was accompanied by corresponding frequency decreases in the other subsets (**Supplementary Figure S4A**).

### 3.5 IgH^MOG^ mice develop bilateral optic neuritis

**Figure 5:**
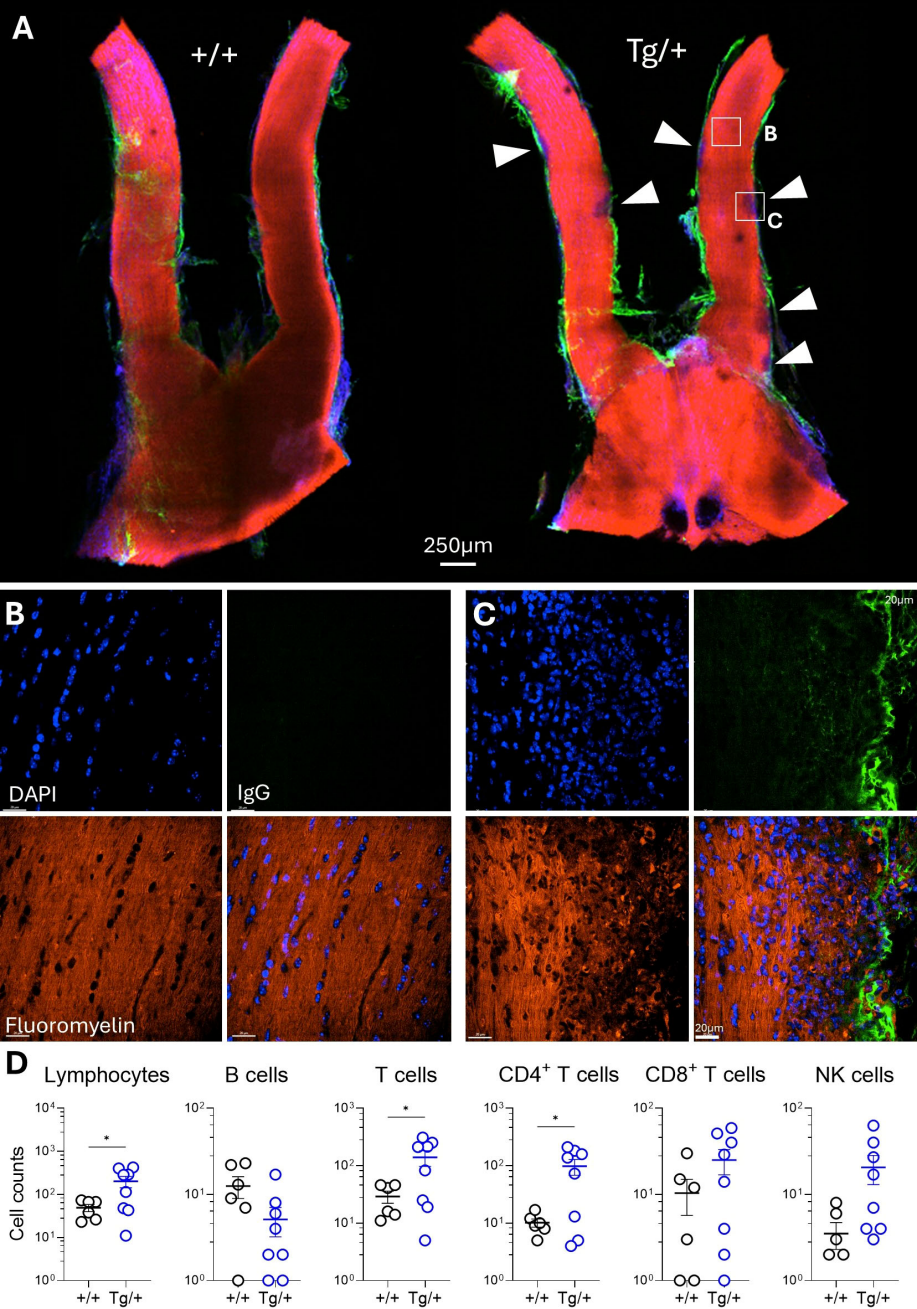
Bilateral optic neuritis in young IgH^MOG^ mice. Five-week-old IgH^MOG^ mice were immunized with MOG_35–55_ without PTX. **(A-C)** Maximal image projections of confocal microscopy at 10X showing whole optic nerves from Tg/+ and +/+ mice at EAE day 14. DAPI is blue, IgG is green, and Fluoromyelin stain is red. Carets highlight lesions **(A)**. Magnification of the boxed areas of the Tg/+ image was imaged at 63X and displayed below **(B, C)**. **(D)** Flow cytometry of optic nerves at EAE day 15, showing total cell count of lymphoid subsets per optic nerve. Gating scheme displayed in **Supplementary Figure S3D**. Proportions of leukocyte subsets in CD45^+^ cells are displayed in **Supplementary Figure S4B**. Data represent one of two similar experiments. Individual animals are represented as points with SEM. Unpaired t-tests were used. *P ≤ 0.05.

Optic neuritis is a key manifestation of MOGAD, so we evaluated the optic nerves in our model. Using whole-mount optic nerves, we performed confocal microscopy. Optic nerves in the Tg/+ mice at day 14 showed DAPI signal-enriched lesions, accompanied by demyelination (**Figures 5A–C**), suggesting optic neuritis. The IgG signal was primarily restricted around the optic nerves, except in lesional areas where faint staining was appreciated within the nerve (**Figure 5C**). Tg/+ mice demonstrated significantly elevated CD4^+^ T cell counts (**Figure 5D**), affecting proportional differences in other cell types (**Supplementary Figure S4B**). Total B cell counts in the optic nerves were not affected.

### 3.6 IgH^MOG^ mice develop brain inflammation

**Figure 6:**
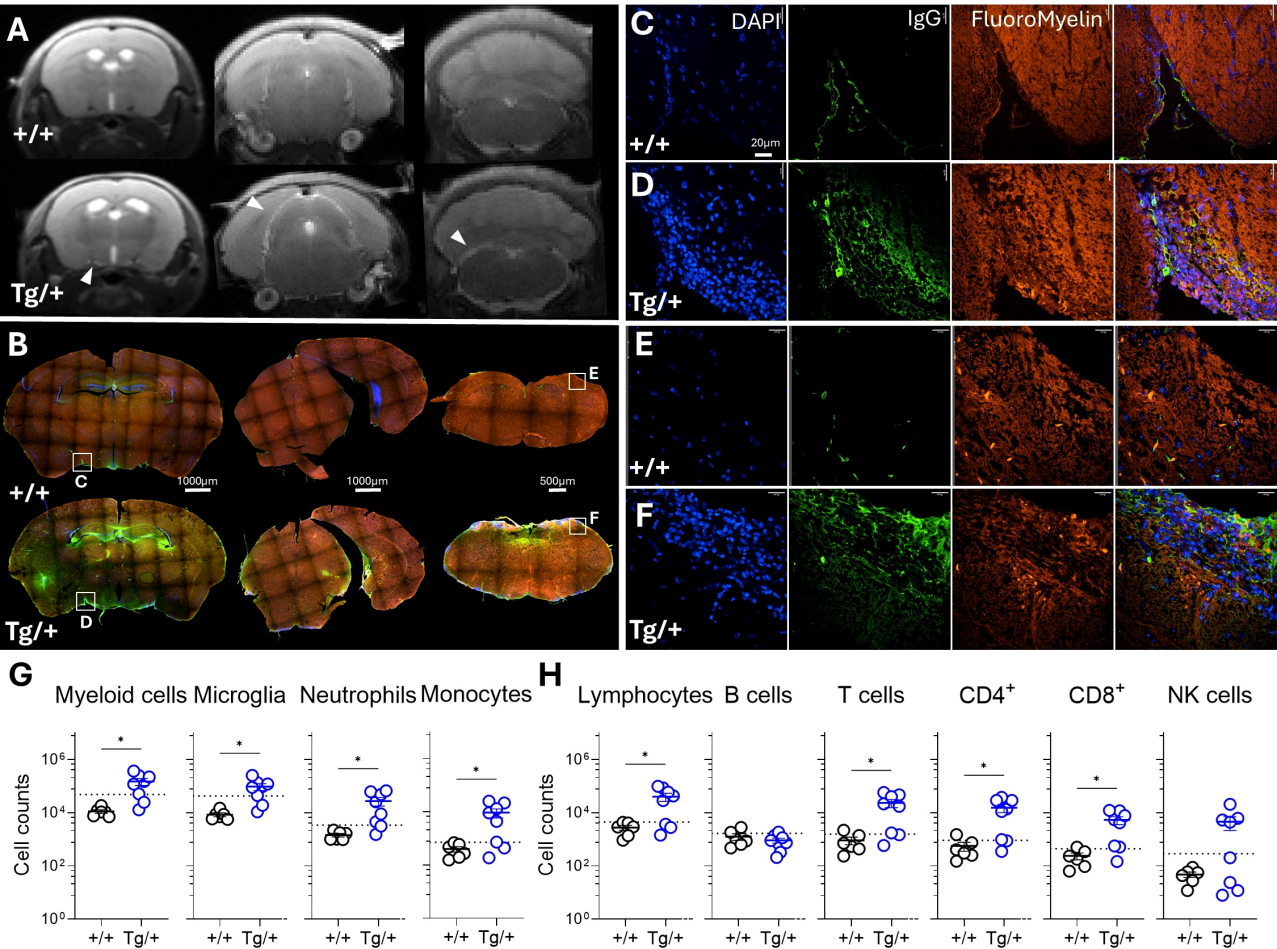
Brain inflammation in young IgH^MOG^ mice. Five-week-old IgH^MOG^ mice were immunized with MOG_35–55_ without PTX. **(A)** Coronal T2-weighted MRI images from live anesthetized animals on EAE day 14. The data represent MRIs from 4 mice. **(B-F)** Confocal microscopy at 10X showing 20 μm coronal sections corresponding to MRI sections at day 14. DAPI is blue, IgG is green, and Fluoromyelin stain is red. Boxed areas were imaged at 63X **(C-F)**. **(G-H)** Flow cytometry of whole spinal cord cells at day 15 after MOG_35–55_ immunization, showing the total number per brain of myeloid **(G)** and lymphoid **(H)** subsets. The dotted line represents values from naïve IgH^MOG^ mice. Gating scheme displayed in **Supplementary Figure S3C**. Proportions of leukocyte subsets in CD45^+^ cells are displayed in **Supplementary Figure S4C**. Data represent one of two experiments. Individual animals are represented as points with SEM. Unpaired t-tests were used. *P ≤ 0.05.

Cerebral and brainstem inflammation are prominent clinical manifestations of MOGAD, especially in pediatrics, so we evaluated these structures in our model. We first performed MRI on Tg/+ compared to +/+ wild-type littermates at day 14. Tg/+ mice had increased T2-weighted signal at the ventral surface of the cerebrum, the interface between the brainstem and the cerebrum, and around the brainstem (**Figure 6A**), suggesting inflammation in those regions. We then performed confocal microscopy on the comparable coronal sections and found that Tg/+ mice showed enhanced IgG staining in the periventricular areas, on the ventral surface of the cerebrum, and at the interface between the brainstem and the cerebrum (**Figure 6B**). Higher magnification imaging of sections from Tg/+ mice revealed lesions on the ventral surface of the cerebrum (**Figure 6C, D**) and circumferentially around the brainstem (**Figure 6E, F**), characterized by rich DAPI staining and IgG deposition. On flow cytometry (**Figure 6G, H**), Tg/+ mice showed increased numbers of myeloid (**Figure 6G**) and lymphoid cells (**Figure 6H**) with significant elevations in the numbers of microglia, neutrophils, monocytes, CD4^+,^ and CD8^+^ T cells. Total B cell numbers were again not different, and shifts in other subset proportions were not significant (**Supplementary Figure S4C**). Increased proportions of Th17 cells in total CD4^+^ T cells were found in the spinal cords and the brains of Tg/+ mice, but the proportion of Th1 cells was not different (**Supplementary Figure S5**).

### 3.7 IgH^MOG^ mice downregulate surface expression of CD16/32 on CNS-infiltrated innate immune cells

**Figure 7:**
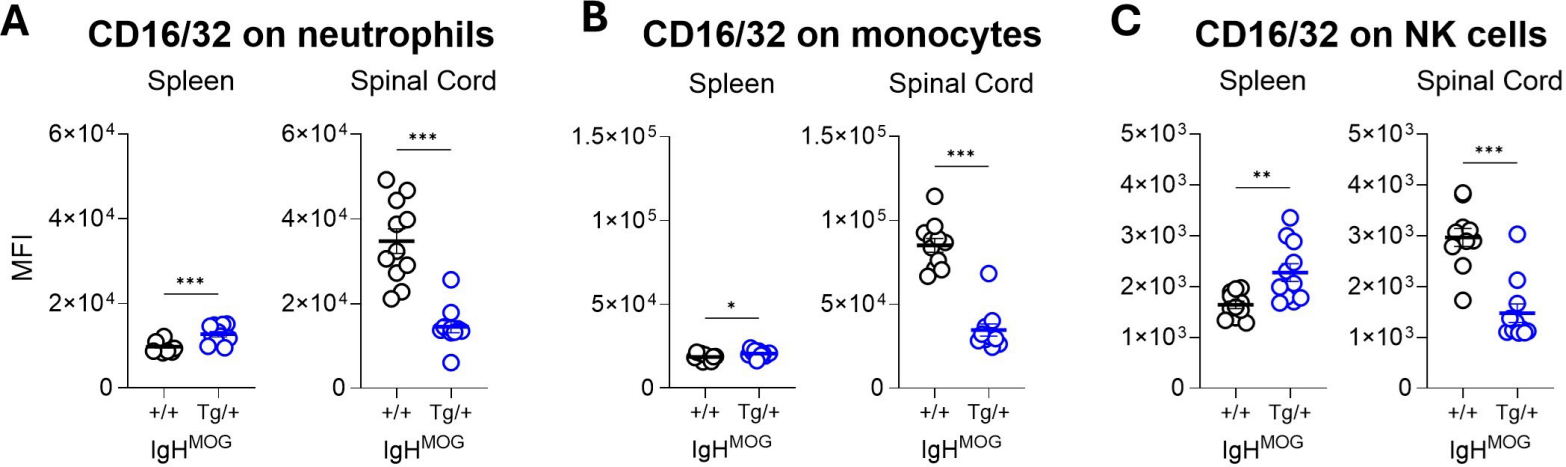
Decreased surface expression of CD16/32 on CNS-infiltrating innate cells in IgH^MOG^ mice. **(A-C)** Five-week-old IgH^MOG^ mice were immunized with MOG_35–55_ without PTX. Flow cytometry was performed on day 15 on the spleen and spinal cord. Mean fluorescence intensity **(MFI)** of CD16/32 surface staining on CD45^hi^CD11b^+^Ly6G^+^ neutrophils **(A)**, CD45^hi^CD11b^+^Ly6C^hi^ monocytes **(B)**, and CD45^hi^CD11b^+^NK1.1^+^ NK cells **(C)**. The data represent one of two experiments. Individual animals are represented as points with SEM. Unpaired t-tests were used. *P ≤ 0.05, **P ≤ 0.01, ***P ≤ 0.001.

Because FcγRs bind the Fc portion of IgG, we evaluated CD16/32 (FcγRIII/II) expression in our model system. In the spleen, Tg/+ mice with MOG immunization showed slightly increased CD16/32 surface expression on neutrophils (**Figure 7A**), monocytes/macrophages (**Figure 7B**), and NK cells (**Figure 7C**), compared to +/+ wild-type littermates. However, in the spinal cord, these innate subsets showed reduced CD16/32 staining in Tg/+ mice compared with wild-type mice, indicating CNS-restricted FcγR involvement. The biological significance of reduced surface CD16/32 expression remains unclear, although a possible explanation emerged when we next examined microglia.

### 3.8 Anti-MOG IgG promotes microglia phagocytosis of MOG

**Figure 8:**
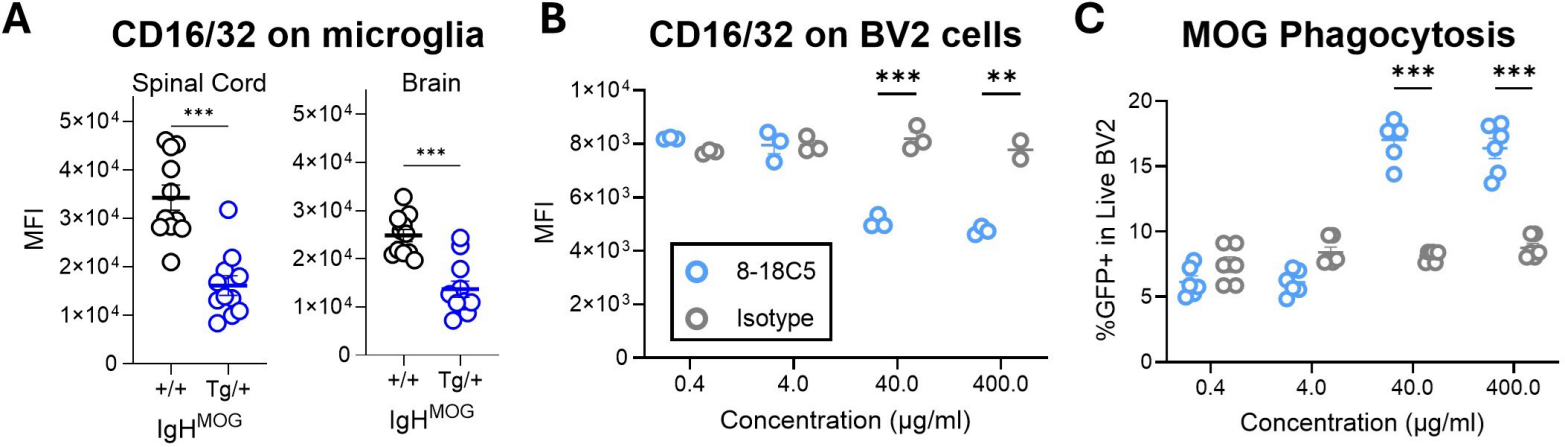
Anti-MOG IgG promotes microglial phagocytosis of MOG-expressing cells. **(A)** Five-week-old IgH^MOG^ Tg/+ mice were immunized with MOG_35–55_ without PTX. Flow cytometry was performed on EAE day 15 on the brain and spinal cord. Mean fluorescence intensity (MFI) of CD16/32 surface staining on CD45^int^CD11b^+^ microglia from spinal cord and brain. **(B-C)** Antibody-dependent phagocytosis assay measuring BV2 uptake of MOG-expressing HEK293T cells that were opsonized with 8-18C5. Flow cytometry staining for surface CD16/32 on live BV2 cells **(B)** and the proportion of live BV2 cells with MOG-turboGFP uptake **(C)**. The data represent one of two experiments. Unpaired t-tests were used. ***P ≤ 0.001.

Similar to the CD16/32 reduction found in CNS-infiltrated cells, we also noted a decrease in CD16/32 staining on microglia in IgH^MOG^Tg/+ compared to +/+ littermates at day 15 (**Figure 8A**). Since FcγR ligation with the Fc regions of antibodies promotes phagocytosis of antibody-opsonized targets, FcγRs would appear down-regulated on the cell surface when internalized together with their ligands (37, 38). Therefore, we hypothesized that MOG antibody enhances microglial phagocytosis of cells expressing membrane-bound MOG in target cells, accompanied by FcγR internalization. This may explain our finding of decreased cell surface CD16/32 staining in Tg/+ microglia. To explore this possibility, we evaluated whether cell surface CD16/32 decreases upon phagocytosis of target cells expressing cell surface MOG in the presence of anti-MOG antibody. To do so, we used MOG-turboGFP-expressing HEK293T cells as phagocytosis targets, opsonized with increasing concentrations of anti-MOG IgG1 (8-18C5). BV2 murine microglia were used as phagocytes. BV2 cells showed decreased MFI of CD16/32 above a threshold concentration of anti-MOG IgG1 (**Figure 8B**). The reduction of CD16/32 surface expression on BV2 cells corresponded to greater phagocytosis of MOG (**Figure 8C**). These results suggested that anti-MOG IgG1 enhances microglial phagocytosis of MOG-expressing cells, which may be one of the pathogenic functions of anti-MOG antibody in MOGAD.

## 4 Discussion

We found that young IgH^MOG^ Tg/+ mice immunized with MOG_35–55_ peptide displayed extensive IgG staining and increased leukocyte infiltration in the spinal cord, optic nerves, and brain. This model also recapitulates pediatric-onset disease using young mice and shows measurable serologic anti-MOG IgG1, as detected by a cell-based assay, the clinical standard. One key difference is that while human MOGAD usually manifests as distinct syndromes such as optic neuritis, transverse myelitis, or ADEM, often across separate attacks, our mouse model showed widespread, concurrent CNS inflammation, revealing the full spatial extent of CNS regions that can be targeted by anti−MOG IgG-mediated inflammation. Exploration of this model revealed the involvement of FcγRs in microglial phagocytosis of MOG-expressing cells, a potential novel mechanism of pathology.

However, we acknowledge several limitations of these murine models for pediatric MOGAD. First, murine IgG1 is functionally distinct from human IgG1, with key differences including greater complement activation, antibody-dependent cellular cytotoxicity, and phagocytosis (39). Second, human anti-MOG IgG1 recognizes different epitopes and binds with lower affinity compared to the mouse anti-MOG IgG1 clone 8-18C5. Human anti-MOG antibodies most commonly target the CC′ loop (40), while 8-18C5 binds to the FG loop on MOG (41). The mouse anti-MOG 8-18C5 antibody also binds to MOG monovalently, whereas human anti-MOG antibodies require bivalent binding (35). Despite these limitations, MOG_35–55_ immunization in young IgH^MOG^ Tg/+ mice that we used in our study still reflects aspects of pediatric MOGAD. One strength of this model is the re-purposing of EAE, which is commonly compared to MS. One long−standing critique of EAE as a model for MS (42) is that EAE is primarily mediated by autoreactive CD4^+^ T cells (43, 44), while MS lesions show a predominance of CD8^+^ T cells (45, 46). In contrast, MOGAD is characterized by CD4^+^ T cell predominance (47, 48), some of which may be MOG-specific (49). Therefore, employing EAE induction in the presence of anti-MOG antibodies may provide mechanistic insights into human MOGAD (50). Human-derived anti-MOG antibodies and B-cell-dependent EAE induction protocols can further improve this model.

Another limitation of this model is that it does not fully recapitulate the resolution and relapse characteristic of a subset of human MOGAD. MRI lesions in patients can resolve spontaneously, but the median time to radiographic resolution is approximately three months (51). Thus, it remains possible that with longer follow−up, some IgH^MOG^ mice might demonstrate a relapsing−remitting pattern. In our study, however, during the one−month observation period, we did not observe clear evidence of remission, although clinical scores declined from their peak. In addition, the standard of care for MOGAD involves treatment of acute attacks with corticosteroids and/or IVIg. Similar interventions in this model might promote remission, but this possibility has not yet been evaluated experimentally. Our findings build on prior studies using the IgH^MOG^ mouse model. Previous work using MOG_35–55_ immunization in adult IgH^MOG^ on an NOD background also observed an earlier onset and more severe EAE in these mice compared to wild-type controls (52). The authors in this study attributed disease exacerbation primarily to the formation of meningeal tertiary lymphoid-like structures and B cell-derived IL-23, which sustained pathogenic Th17 responses. Consistent with these findings in NOD background mice, we also observed enrichment of Th17 responses in the CNS of young IgH^MOG^ mice on the B6 background. However, their study did not find that their IgH^MOG^ mice had higher levels of serum anti-MOG IgG than wild-type mice, as measured by ELISA using *E. coli*-derived MOG_1-125,_ which cannot detect antibodies binding to conformational MOG. Natively folded MOG as an assay substrate is critical for detecting conformation-specific human anti-MOG antibodies (34). Based on this clinical insight, we employed a cell-based assay using the full-length MOG protein expressed by mammalian cells, which is the clinical standard for the diagnosis of MOGAD. In contrast to the ELISA, this cell-based assay showed significantly higher levels of serum anti-MOG IgG1 in IgH^MOG^Tg/+ compared to wild-type littermate mice. Notably, we identified that maternal anti-MOG IgG is carried over to wild-type progeny and plays a role in the progeny’s EAE disease. Spontaneous EAE onset after weaning was reported in progeny from the crossbreeding of 2D2 and IgH^MOG^ mice (18, 19), but the data showed high variability in disease incidence, ranging from 0% to 96% across litters (53). Based on our findings, the progeny’s propensity for developing spontaneous EAE may depend on whether the mating scheme used IgH^MOG^ dams to allow for maternal anti-MOG antibodies to be transmitted.

Emerging evidence implicates FcγR engagement and antibody-dependent phagocytosis in the pathogenesis of MOGAD, a concept supported by our experimental observations. Spatola et al. surveyed sera from 123 patients with MOGAD and identified two distinct endophenotypes (54). The “pro-inflammatory” endophenotype was enriched in children with active disease and characterized by elevated MOG-specific antibody titers, enhanced CD16/32 (FcγRII/III) binding capacity, and activation of NK cells. The alternative endophenotype, characterized by attenuated FcγR engagement and lower anti-MOG antibody titers, was associated with disease remission (54). Schmid et al. also revealed aberrations in FcγR staining in NK cells, monocytes, and dendritic cells in patients with MOGAD. In particular, they found that NK cells reduced surface CD16 (FcγRIII) staining in MOGAD, similar to other IgG1-mediated autoimmune diseases, such as myasthenia gravis and NMOSD (55). Yandamuri et al. demonstrated that serum from patients with MOGAD induced antibody-dependent phagocytosis of MOG-expressing cells by THP-1 macrophages (14). Additionally, perivascular MOG-laden macrophages are a characteristic pathological finding in MOGAD (48). Paralleling these human observations, our MOGAD model showed downregulation of CD16/32 (FcγRII/III) on innate cells, including microglia. We further demonstrated that microglia downregulate surface FcγR concomitantly with MOG antibody-dependent phagocytosis of MOG-expressing cells. Given the ever-expanding discovery of CNS-specific autoantibodies, future studies examining the consequences of antibody−mediated phagocytosis of CNS antigens by microglia may provide insight into this potentially convergent pathway in neurologic autoimmune diseases.

In summary, our pediatric MOGAD models provide a clinically relevant framework for dissecting the pathogenesis of autoantibody-mediated neuroinflammation. Employment of cell-based assays for detecting anti-MOG IgG and the use of IgH^MOG^ Tg sires, not dams, are critical to establishing this model and its relevance to human disease. Our findings also suggest that anti−MOG IgG engages FcγRs and promotes phagocytosis of MOG-expressing cells by microglia and other phagocytes in the CNS. Continued refinement of this model system to better reflect human aspects of disease may help unravel immunopathogenesis to inform patient care.

## Data Availability

The raw data supporting the conclusions of this article will be made available by the authors, without undue reservation.
