# Supplementary material for: Anti-MOG IgG in EAE models clinical aspects of pediatric MOGAD
Source: Front Immunol. 2026 Jun 15;17:1860892. doi: 10.3389/fimmu.2026.1860892 (PMC13310711; doi:10.3389/fimmu.2026.1860892)
Supplement: Supplementary file 1 [file DataSheet1.pdf]

Supplementary Material

SFIG1

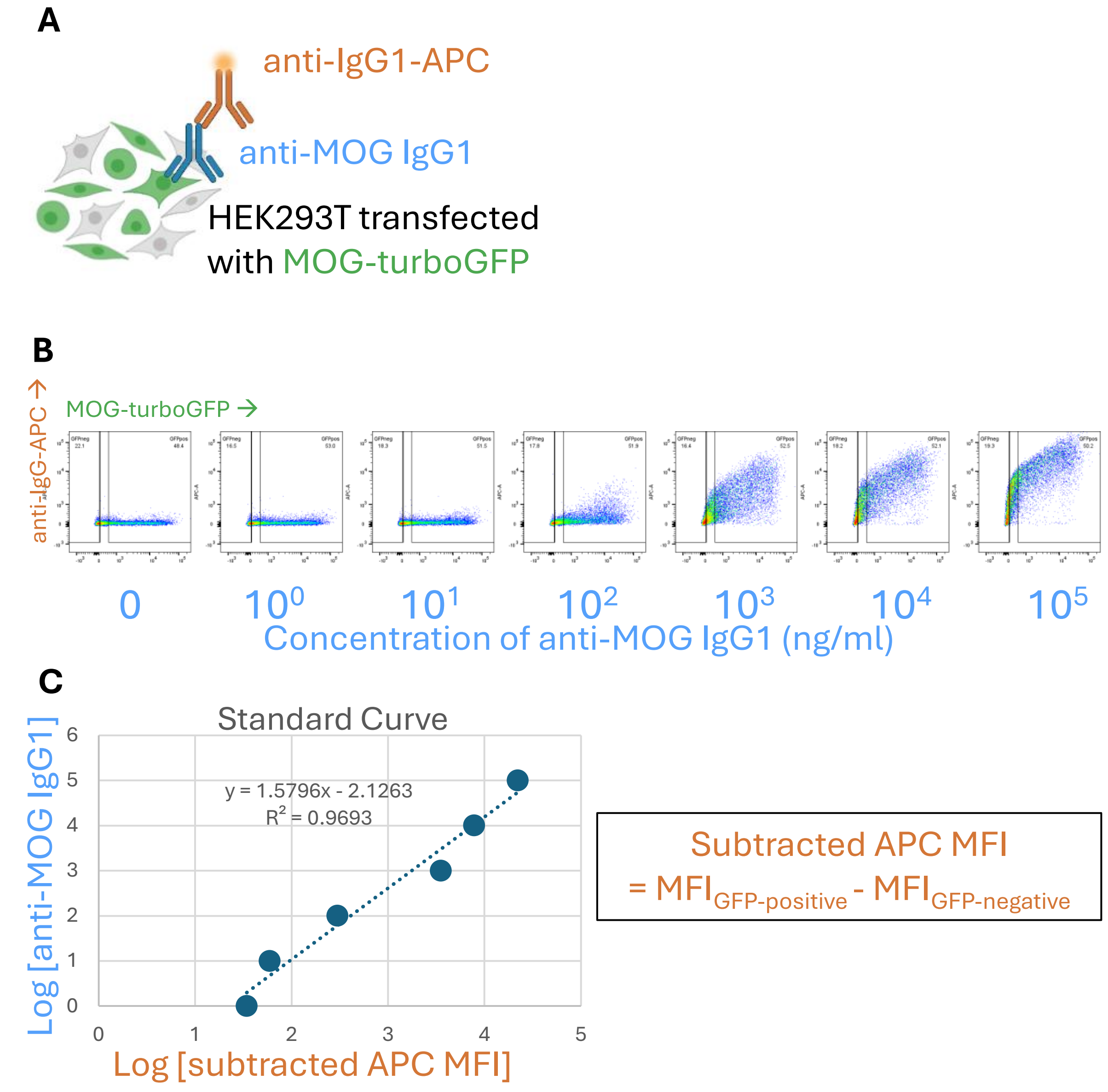

Figure S1: Flow cytometric cell-based assay for conformation-specific anti-MOG IgG. (A) Assay schematic. HEK293T cells are transfected with the mouse MOG-turboGFP expression vector. Cells are incubated with serum containing anti-MOG IgG1, which is detected with a fluorochrome-conjugated secondary antibody. **(B)** Sample flow cytometric data with increasing concentration of murine 8-18C5. **(C)** Sample standard curve used for quantification of anti-MOG IgG1 concentration.

SFIG2

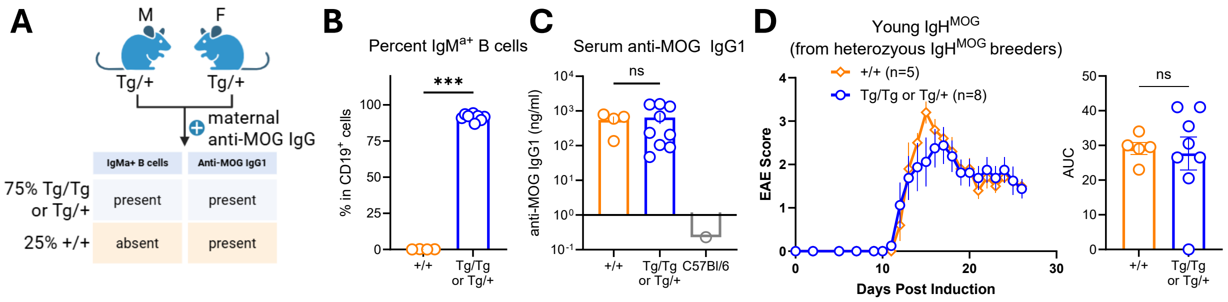

Figure S2: Maternal anti-MOG antibodies impact disease development in progeny. (A) Breeding scheme with heterozygous IgH^{MOG} Tg mice. **(B, C)** Resultant progeny's proportion of congenically marked IgM^{a}+ anti-MOG B cells in the blood (B) and serum anti-MOG IgG1 levels (C) at weaning. **(D)** Mice were immunized with MOG_{35-55} as described in the methods, along with 200 ng of PTX on days 0 and 2. Lines represent averages of EAE disease scores with SEM. Unpaired t-test was used.

SFIG3

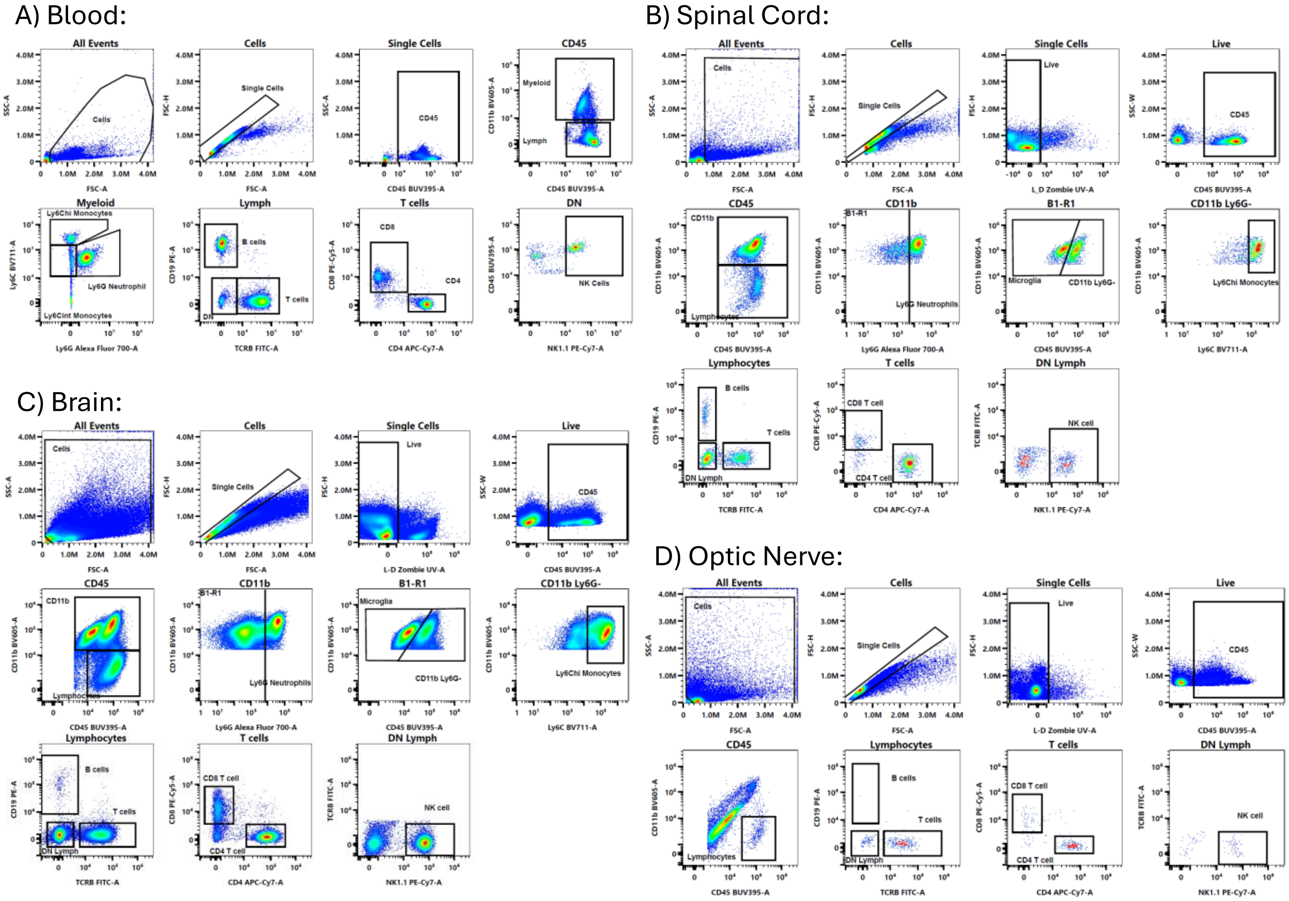

Microglia: CD45^{int} CD11b^{+}

Myeloid: CD45^{hi}CD11b^{+}

Neutrophils: CD45^{hi}CD11b^{+}Ly6G^{+}

Monocytes: CD45^{hi}CD11b^{+}Ly6C^{hi}

Lymphocytes: CD45^{hi}CD11b^{-}

NK cells: CD45^{hi}CD11b^{-}CD19^{-}TCRβ^{-}NK1.1^{+}

B cells: CD45^{hi}CD11b^{-}CD19^{+}TCRβ^{-}

T cells: CD45^{hi}CD11b^{-}CD19^{-}TCRβ^{+}

CD4^{+} T cells: CD45^{hi}CD11b^{-}CD19^{-}TCRβ^{+}CD4^{+}

CD8^{+} T cells: CD45^{hi}CD11b^{-}CD19^{-}TCRβ^{+}CD8^{+}

Figure S3: Spectral flow cytometric gating strategy. Flow panels and gating strategies for blood (A), spinal cord (B), brain (C), and optic nerves (D) are run on the Cytek Aurora and analyzed in SpectroFlo software.

SFIG4

A) Spinal Cord Flow Proportions: Figure 4 companion

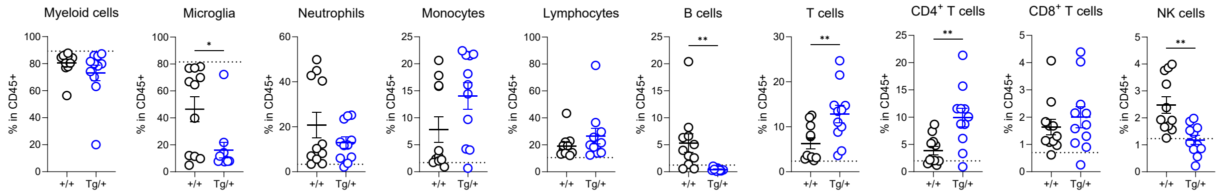

B) Optic Nerve Flow Cytometry Proportions: Figure 5 companion

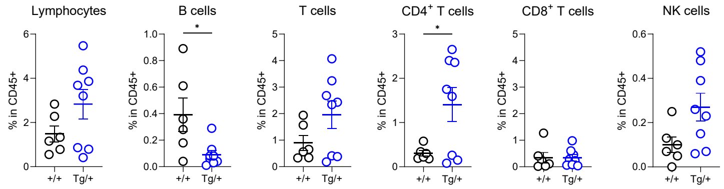

C) Brain Flow Cytometry Proportions: Figure 6 companion

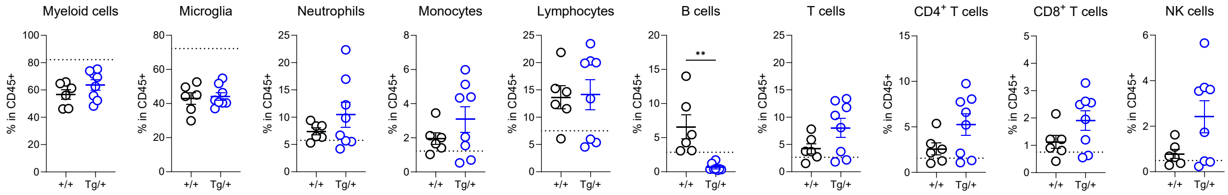

Figure S4: CNS flow cytometry with percentages of CD45^{+}. These graphs correspond to Figure 4 (A), Figure S3 (B), and Figure 5 (C).

SFIG5

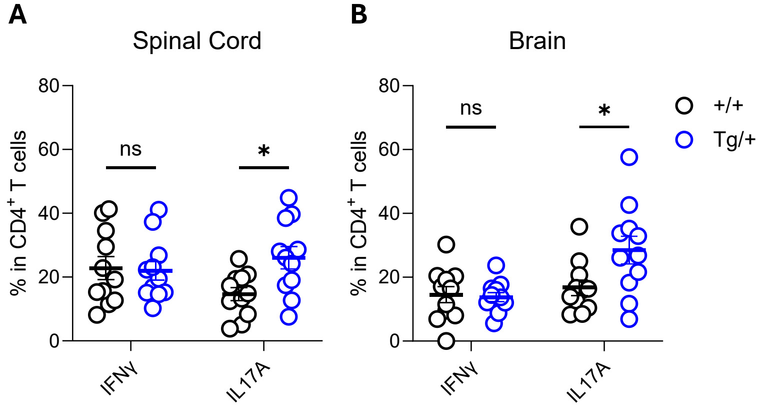

Figure S5: Th1 and Th17 cell detection in the CNS of IgH^{MOG} Tg mice. Intracellular cytokine staining of cells from spinal cords (A) and brains (B) at day 15 after MOG_{35-55} immunization. Individual animals are represented as points with SEM. Unpaired t-test was used.
